# Elimination of ^15^N-thymidine after oral administration in human infants

**DOI:** 10.1371/journal.pone.0295651

**Published:** 2024-01-25

**Authors:** Niyatie Ammanamanchi, Jessie Yester, Anita P. Bargaje, Dawn Thomas, Kathryn C. Little, Shannon Janzef, Kimberly Francis, Jacqueline Weinberg, Jennifer Johnson, Thomas Seery, Tyler Hutchinson Harris, Bryan J. Funari, Kirsten Rose-Felker, Matthew Zinn, Susan A. Miller, Shawn C. West, Brian Feingold, Hairu Zhou, Matthew L. Steinhauser, Timothy Csernica, Robert Michener, Bernhard Kühn

**Affiliations:** 1 Division of Pediatric Cardiology, Pediatric Institute for Heart Regeneration and Therapeutics (I-HRT), UPMC Children’s Hospital of Pittsburgh, Pittsburgh, PA, United States of America; 2 Department of Pediatrics, University of Pittsburgh School of Medicine, Pittsburgh, PA, United States of America; 3 Clinical Research Support Services (CRSS), Department of Pediatrics, UPMC Children’s Hospital of Pittsburgh, Pittsburgh, PA, United States of America; 4 UPMC Heart and Vascular Institute, UPMC Presbyterian, Pittsburgh, PA, United States of America; 5 Aging Institute, University of Pittsburgh, Pittsburgh, PA, United States of America; 6 Division of Chemistry and Chemical Engineering, California Institute of Technology, Pasadena, CA, United States of America; 7 Department of Biology, Boston University Stable Isotope Laboratory, Boston, MA, United States of America; 8 McGowan Institute of Regenerative Medicine, University of Pittsburgh School of Medicine, Pittsburgh, PA, United States of America; Foshan University, CHINA

## Abstract

**Background:**

We have developed a new clinical research approach for the quantification of cellular proliferation in human infants to address unanswered questions about tissue renewal and regeneration. The approach consists of oral ^15^N-thymidine administration to label cells in S-phase, followed by Multi-isotope Imaging Mass Spectrometry for detection of the incorporated label in cell nuclei. To establish the approach, we performed an observational study to examine uptake and elimination of ^15^N-thymidine. We compared at-home label administration with in-hospital administration in infants with tetralogy of Fallot, a form of congenital heart disease, and infants with heart failure.

**Methods:**

We examined urine samples from 18 infants who received ^15^N-thymidine (50 mg/kg body weight) by mouth for five consecutive days. We used Isotope Ratio Mass Spectrometry to determine enrichment of ^15^N relative to ^14^N (%) in urine.

**Results/findings:**

^15^N-thymidine dose administration produced periodic rises of ^15^N enrichment in urine. Infants with tetralogy of Fallot had a 3.2-fold increase and infants with heart failure had a 4.3-fold increase in mean peak ^15^N enrichment over baseline. The mean ^15^N enrichment was not statistically different between the two patient populations (p = 0.103). The time to peak ^15^N enrichment in tetralogy of Fallot infants was 6.3 ± 1 hr and in infants with heart failure 7.5 ± 2 hr (mean ± SEM). The duration of significant ^15^N enrichment after a dose was 18.5 ± 1.7 hr in tetralogy of Fallot and in heart failure 18.2 ± 1.8 hr (mean ± SEM). The time to peak enrichment and duration of enrichment were also not statistically different (p = 0.617 and p = 0.887).

**Conclusions:**

The presented results support two conclusions of significance for future applications: (1) Demonstration that ^15^N-thymidine label administration at home is equivalent to in-hospital administration. (2) Two different types of heart disease show no differences in ^15^N-thymidine absorption and elimination. This enables the comparative analysis of cellular proliferation between different types of heart disease.

## Introduction

Cellular proliferation is a fundamental mechanism of tissue growth and regeneration, however quantifying cellular proliferation in humans is challenging. Radiolabels, fluorescent compounds, and halogenated analogs have all been invaluable tools to quantify proliferation but have the potential to impact the pathway being studied, and also have the potential for toxicity. More recently, an alternative approach to quantify cellular proliferation using administration of stable isotopes coupled with Multi-isotope Imaging Mass Spectrometry has been developed [1, 2]. Published studies in adult humans have utilized intravenous ^15^N-thymidine or ^2^H_2_O to mark cells in S-phase [3]. Incorporation of ^15^N-thymidine can then be visualized in tissue samples with Multi-isotope Imaging Mass Spectrometry (MIMS, this methodology combines isotopic labeling with tissue analysis by Imaging Mass Spectrometry). We have modified this approach to administer ^15^N-thymidine orally [2].

We have previously reported the quantification of endogenous heart muscle cell (cardiomyocyte) proliferation using oral ^15^N-thymidine administration in one infant [4]. However, the pharmacokinetics of oral ^15^N-thymidine absorption and elimination are unknown. Here, we report the uptake and elimination of ^15^N-thymidine in a series of infants. Although this research study involves human subjects, it does not constitute a clinical trial, as there was no intervention to augment proliferation.

In ^15^N-thymidine, nitrogen atoms in the thymine pyrimidine ring contain an additional neutron, hence, ^15^N. Thymine is not known to have a significant first-pass effect or salvage pathways, i.e., most cells do not rapidly break it down to incorporate it into their metabolism. Thus, ^15^N can be measured as proxy for the administered ^15^N-thymidine. The abundance of ^15^N can be measured by quantifying the heavy ^15^N relative to the abundant light ^14^N with Isotope Ratio Mass Spectrometry (IRMS) [1, 3, 5–7]. Junk *et al*. found the absolute ratio of ^14^N/^15^N in atmospheric nitrogen to be 272.0 ± 0.3 [8]. Thus, the ^15^N/^14^N atomic ratio (i.e., the ratio of all ^15^N atoms to all ^14^N atoms) is 0.0037 (0.37%). Physiological ^15^N/^14^N ratios in the absence of labeling show little variation at this level of precision. Kraft *et al*. investigated this ratio in the blood of 206 patients reporting values of 0.3708 ± 0. 0002%, while multiple studies of urine [9, 10] have found ^15^N/^14^N values ranging from 0.3685 to 0.3712%. [11]. Cells with an increased ^15^N/^14^N ratio in the nucleus, assessed by MIMS, are cells that have undergone DNA synthesis when the available ^15^N-thymidine was sufficiently enriched.

Stable isotopes are safe for use in human studies. ^15^N-labeled amino acids have been used in over 95 human studies, including one study of catabolism and nitrogen turnover in infants, as early as the 1969 [12–14]. Thymidine is considered safe for administration to infants in the doses described by this study and does not require approval by the Federal Drug Administration (FDA) of the United States [2, 15].

In humans, intravenous administration of stable isotopes has been used to quantify cellular proliferation. However, intravenous administration requires inpatient admission and is difficult to justify in pediatric patients. Therefore, this administration mode limits the types of questions that could otherwise be addressed with the method. The MIMS approach was modified to facilitate the quantification of proliferation in pediatric patients using an oral label [2], however the pharmacodynamics are undefined. To close this knowledge gap, we have considered that the uptake and elimination of administered thymidine can be studied by analyzing urine samples [16]. This avoids serial blood draws, which are difficult to justify in pediatric patients and are impractical in out-of-hospital settings. Infants void up to once per hour [17], thus providing the opportunity to obtain urine samples frequently, albeit at unpredictable intervals. Urine concentration can vary based on the hydration status of the patient; to circumvent this variable, we analyzed the non-volatile part of urine for a more consistent way to quantify ^15^N elimination.

While the MIMS methodology is broadly applicable to study cellular proliferation, our application was designed to quantify cardiomyocyte proliferation in discarded pieces of myocardium. We designed the approach of one administration per day for five consecutive days with consideration for family compliance and need for longer duration of label administration to detect rare cardiomyocyte cell cycle events. We selected two patient groups for this initial study: infants with tetralogy of Fallot (ToF) and infants with heart failure (HF). ToF is a form of congenital heart disease that requires surgical repair, often performed around 3–6 months after birth. The repair frequently requires resection of myocardium that would otherwise be discarded. The availability of myocardial samples at predictable ages makes this patient population appropriate for the study of cardiomyocyte proliferation. Infants with ToF typically do not develop heart failure and grow normally. As such, the elimination results obtained in this population should be broadly applicable. Our second study group included infants with severe heart failure who were listed for heart transplantation. Patients with HF have altered energy expenditure, which may impact ^15^N-thymidine uptake and excretion [18]. Because of the severity of their heart failure, these patients were in the hospital, which enabled ^15^N-thymidine administration and urine sample collection under more controlled conditions. We have compared the elimination patterns after oral ^15^N-thymidine administration between the two patient populations: ToF and HF. Our results demonstrate the uptake and elimination of ^15^N-thymidine is stable across our target patient populations.

## Methods

### Study approval

In this observational pilot study, research participants did not receive interventions to augment cellular proliferation, as such, this study is not a clinical trial. The human research protocol was reviewed and approved by Institutional Review Board (IRB, STUDY19030250, University of Pittsburgh). The approval dates are summarized in **S1 Table**.

#### Study design

This study was designed as an observational pilot study and funded by the National Heart, Lung, and Blood Institute (NHLBI) of the USA as an observational study in humans. Because there were no interventions to modulate cardiomyocyte proliferation, the NHLBI did not classify this design as clinical trial. Because of the high probability of yielding resected myocardium during their corrective heart surgeries, infants with ToF or ToF variant of double-outlet right ventricle (DORV) were screened for study enrollment. To enroll infants with heart failure, infants with any cardiac diagnoses who were candidates for transplantation were eligible to participate. Recruitment of patients and study occurred between 07-23-2015 to 11-14-2022. About 124 patients were assessed for eligibility, thirteen families with infants having a diagnosis of ToF or ToF variant of DORV, four families with infants with DCM, and one family with an infant with HLHS gave informed and written consent to participate. Caregivers of ToF infants that met enrollment criteria were given instructions for administering oral ^15^N-thymidine, collecting urine samples, and filling out documentation logs at home. Study patients received 50 mg/kg of ^15^N-thymidine once daily for 5 days. Only one ToF patient received ^15^N-thymidine while in the hospital. HF infants with DCM or HLHS were admitted during their five-day ^15^N-thymidine administration period. Hospital personnel performed the same tasks that were performed by caregivers. Caregivers, including hospital personnel, received paper logs to enter dates and times of ^15^N-thymidine administrations and urine collections over the five-day labeling period. Cotton balls were placed in diapers and collected after infants voided. Urine-saturated cotton balls were collected after the first ^15^N-thymidine dose up until 24 hours after the last dose. Urine samples were collected beginning 09-06-2016 to 11-20-2022. To avoid conflicts of interest, the protocol did not include any rewards for collected urine samples or returned logs.

#### Identification of patients whose data were suitable for systematic analysis

In this study we have included data from any patient whose caregiver administered ^15^N-thymidine, returned a urine collection log and who had at least one urine sample collected after ^15^N-thymidine administration. Out of 18 families, 14 families returned urine samples and urine collection logs. Three of the 14 families did not record urine collections and/ or ^15^N-thymidine administration times and were excluded from further analysis. A total of 262 urine samples, from 11 patients, were available for analysis.

#### MIMS analysis

The clinical study was approved by the IRB, parents have given the informed consent, and the MIMS-protocol as described in Yester et. al, 2021 [2] was followed. In brief, at 3.5 weeks after birth, the patient received ^15^N-thymidine at the time of consent [50 mg/kg per os (po), for five consecutive days], followed by ToF surgery at 7 months. A piece of tissue from right ventricular myocardium was collected and fixed in 4% paraformaldehyde. The tissue was embedded in LR White, followed by sectioning (500 nm) and mounting on silicon chips. Myocardial sections were placed in a NanoSIMS 50L (CAMECA) for MIMS analysis. Measurement of ^15^N-thymidine labeling was quantified using the ^12^C^15^N^−^/^12^C^14^N^−^ ratio, which was obtained from mass images (^12^C^14^N^−^, ^31^P and ^32^S). Images were then analyzed using OpenMIMS version 3.0, ImageJ (NIH) available at https://github.com/BWHCNI/OpenMIMS. The cellular identity was quantified by using the ^14^N (^12^C^14^N^−^) images. Identification of cardiomyocyte nuclei was confirmed based on the close association with sarcomeric structures.

#### Processing of urine samples

Control urine samples (infants who did not receive ^15^N-thymidine administration): To establish baseline and the variation of ^15^N/^14^N in the absence of administration of any external ^15^N, we have examined the urine from five infants who did not receive ^15^N-thymidine. The discarded urine was obtained from five patients in a de-identified fashion from the Children’s Hospital of Pittsburgh Clinical Laboratory. Age of the patients was <6 months, i.e., matched to the age of the study patients. None of the patients had evidence for urinary tract infection (UTI).

#### Recording and processing of urine samples from patients who received ^15^N-thymidine administration

Time of ^15^N-thymidine administration was recorded in paper logs. After ^15^N-thymidine cotton balls were placed in the infant’s diaper for urine collection. When diapers were removed, caregivers or study coordinators recorded the time of urine collection times directly on specimen collection bags and on provided paper logs. Time of ^15^N-thymidine administration was recorded in paper logs. The documented times written on specimen bags included date, hours, and minutes. The paper logs had collection times approximated to the nearest hour by caregivers. All dates and times written on specimen collection bags were cross-checked with log recordings and utilized in analysis. When available, we utilized data recorded with greater accuracy i.e., time recorded as day: hour: minute. Discrepancies between specimen bag recordings and paper log recordings were reconciled in the context of all available information. For instance, if raw data recorded by caregivers was biologically or realistically impossible, i.e., time recorded occurred before supplies to collect urine was provided to family, careful reconciliation occurred. Urine-saturated cotton balls were placed in specimen bags and stored at -80˚C. Cotton balls were placed into 5 mL syringes and liquid was squeezed out into 1.5 mL Eppendorf tubes. Tubes were kept at– 20 ˚C. On average, 1.1 mL ± 0.4 mL of urine (n = 262, range 0.5 mL to 1.5 mL) was expressed per cotton ball and an average of 19 urine samples were collected per test infant.

#### Isotope ratio mass spectrometry (IRMS) of control samples

Tin capsules were weighed on a Sartorius microbalance. The urine sample was then pipetted into the capsule, which was then placed in a 96 well tray. The tray was put in a 60 ˚C drying oven overnight. The capsule was weighed again, and the difference between the empty capsule and the dried capsule was recorded as the mass of the dried urine sample. Dried urine weights were between 1.6 to 4.5 mg. The samples were shipped to the Center for Stable Isotopes at the University of New Mexico. Nitrogen and carbon isotope ratios were measured by Elemental Analyzer Continuous Flow Isotope Ratio Mass Spectrometry using a Costech ECS 4010 Elemental Analyzer coupled to a ThermoFisher Scientific Delta V Advantage mass spectrometer via a CONFLO IV interface. Analyses were normalized to internal laboratory standards run at the beginning, at intervals between samples, and at the end of laboratory sessions. The ratio of ^15^N/^14^N (%) was expressed as the relative per mil (‰) difference between the samples and the international standard (N_2_ in air R_AIR_ = 0.003676), where: δ^15^N_AIR_ = (R_sample_/ R_AIR_-1) x 1000 (‰) and R = ^15^N/^14^N; atom percents were calculated from these values. The standards were: UNM-CSI Protein std #1 (casein purchased from Sigma Aldrich with δ^15^N_AIR_ values of 6.43 ‰; UNM-CSI Protein std#2 (soy protein purchased from Sigma Aldrich with δ^15^N_AIR_ values of 0.98 ‰; and UNM-CSI protein Std#4, (house made tuna protein with δ^15^N_AIR_ values of 13.32 ‰). These standards were calibrated against the ^15^N standards IAEA N_1_, IAEA N_2_ and USGS 43. for δ^15^N.

#### Isotope ratio mass spectrometry (IRMS) of test samples

Dried urine samples were prepared in tin capsules as described above. Dried urine weights were between 0.10 to 6.03 mg per sample. The samples were shipped to Boston University stable isotope laboratory. The samples were then combusted in an Elementar Cube elemental analyzer. The combustion gases (N_2_ and CO_2_) were separated, with CO_2_ collected and N_2_ gas passed through a GVI (GV Instruments, Wythenshave, Manchester, United Kingdom) diluter and reference gas box, and introduced into the GVI IsoPrime isotope ratio mass spectrometer. Those values were then blank-corrected using a size series of glycine [19]. Those isotope values were corrected using a 2-point normalization of U.S. Geological Survey standards (USGS 40 and 41). Because the samples were labeled with enriched ^15^N, the values were expressed as atom percent. Analysis with IRMS was performed in 3 batches. The results (in atomic %) of the controls for the first batch were glycine 0.371 ± 0.001 (n = 27) and peptone 0.371 ± 0.010 (n = 13), for the second batch glycine 0.371 ± 0.001 (n = 15) and peptone 0.369 ± 0.010 (n = 7), and for the third batch glycine 0.371 ± 0.001 (n = 4) and peptone 0.369 ± 0.010 (n = 3).

#### ^15^N enrichment levels analysis of ToF and HF urine samples

The ^15^N/^14^N atomic ratio were plotted using Prism 9 (GraphPad). The x-axes indicate the hours at which the urine samples were collected, and the y-axes represent the corresponding ^15^N/^14^N (%) in urine samples. The x-axis begins at 00:00 hours. The vertical green arrows signify the time at which doses of ^15^N-thymidine were administered. All chronological ^15^N/^14^N (%) data was connected by solid black lines unless successive urine collections occurred > 24 hrs apart. Gaps in urine collection hinder ^15^N/^14^N (%) trend expectations related to dose administrations. The ^15^N/^14^N (%) of the controls (0.369 ± 0.001 ≈ 0.37%) was indicated in horizontal red lines to assist in visualization of ^15^N enrichment during the 5-day labeling period.

#### Statistical analysis–fitting the data

To determine when maximum and minimum urine elimination occurs, we have fitted the data. Urine sample collection time points were not uniformly spaced because they were collected after spontaneous voids. For the same reason, different patients have different numbers of samples. These features of the data sets represent challenges for the identification of appropriate fitting models. Given these goals and constraints, we have considered the following methods: AutoRegressive Integrated Moving Average (ARIMA), Locally Estimated Scatterplot Smoothing (LOESS), and polynomial. ARIMA is a time-series analysis, which assumes successive equally spaced points in time. Because our urine samples are not equally spaced in time, this method would not be appropriate. LOESS is a non-parametric fitting method. However, this flexible smoothing approach could lead to very different trajectories between individuals, while a third-degree polynomial ensures functional similarities between all patients’ estimated trajectories. Given these considerations, it was determined that the polynomial approach was the most appropriate statistical model for our data. We compared different degrees of polynomial fitting, which identified 3^rd^ order polynomial as most appropriate (**S1 Fig**).

All fitting curves were reported as mean ± 2 SD (95% CI). Curve fits and statistical tests were performed with Prism 9 (GraphPad). To prevent underfitting the data (lower degree polynomial; high bias) and overfitting the data (higher degree polynomial; high variance) the optimal degree of the polynomial was derived from the determination of the model with the lowest polynomial complexity and highest R^2^ value. Using GraphPad Prism, different degrees of polynomial functions were tested to determine the best nonlinear fitting function. Centered polynomial functions were used to avoid high covariance and dependency typical of regular polynomial functions. Centering by subtracting the mean x value from all x values prevent large standard errors, wide confidence intervals, and wide confidence or prediction bands. Centered first, second, third, fourth, fifth, and sixth-degree polynomials were compared in terms of function simplicity and R^2^ value (**S2 Table**). The R^2^ value appeared to plateau at a maximum value for third degree polynomials and did not significantly increase for higher order polynomials tested. For this reason, we selected the centered third-degree polynomial given its function simplicity and high R^2^ value (**S1 Fig**). While the third-degree polynomial may not be the best fit according to R^2^ value for all our patients, it is the best fit for many of them (ideally, most). Because we should fit the results from different patients with the same mathematical model, we chose the centered third-degree polynomial model.

#### Statistical analysis–quantifying peak ^15^N enrichment and labeling period

All graphs were fit with centered third-degree polynomial functions and mean ± 2 SD (95% CI) lines were plotted. Peak ^15^N enrichment values and times are labeled on graphs as dashed red lines. All calculations and transformations to acquire ToF and HF comparison graphs were also performed with Prism 9 (GraphPad). Corresponding results are reported as mean ± SEM (%). Peak and minimum enrichment was calculated as the highest and lowest ^15^N/^14^N (%) respectfully. The first derivative of the third-degree polynomial fitting curve was taken followed by determination of all first derivative (x,y) coordinates for which y = 0. Corresponding x-values were taken as times of maximum enrichment and minimum enrichment respectfully. These x-values were used to calculate corresponding y-values on original third-degree polynomial curves. The y-values represent peak enrichment and minimum enrichment respectfully. Labeling period was defined as the duration of time over which the lower 95% CI remained above the baseline ^15^N/^14^N (%) of 0.37% (negative control). If the lower 95% CI > 0.37% at time point 0, the labeling interval began at 0 hours. If the lower 95% CI < 0.37% at time point 0, the labeling interval began at the time when the lower 95% CI = 0.37%. The labeling period ended when the lower 95% CI dropped below 0.37%. If the lower 95% CI remained above 0.37% until the end of the labeling day, the labeling period ended at the time of minimum enrichment. The statistical analysis consisted of comparing between sample populations using unpaired t-tests with Welch’s correction. All p-values are reported as two-tailed. Mean enrichment ± SEM, mean of peak enrichment ± SEM, mean of peak enrichment timing ± SEM, and mean labeling period ± SEM in each patient population (ToF / HF) was calculated and graphed. We defined the threshold for significance as α<0.05.

## Results

**Fig 1:**
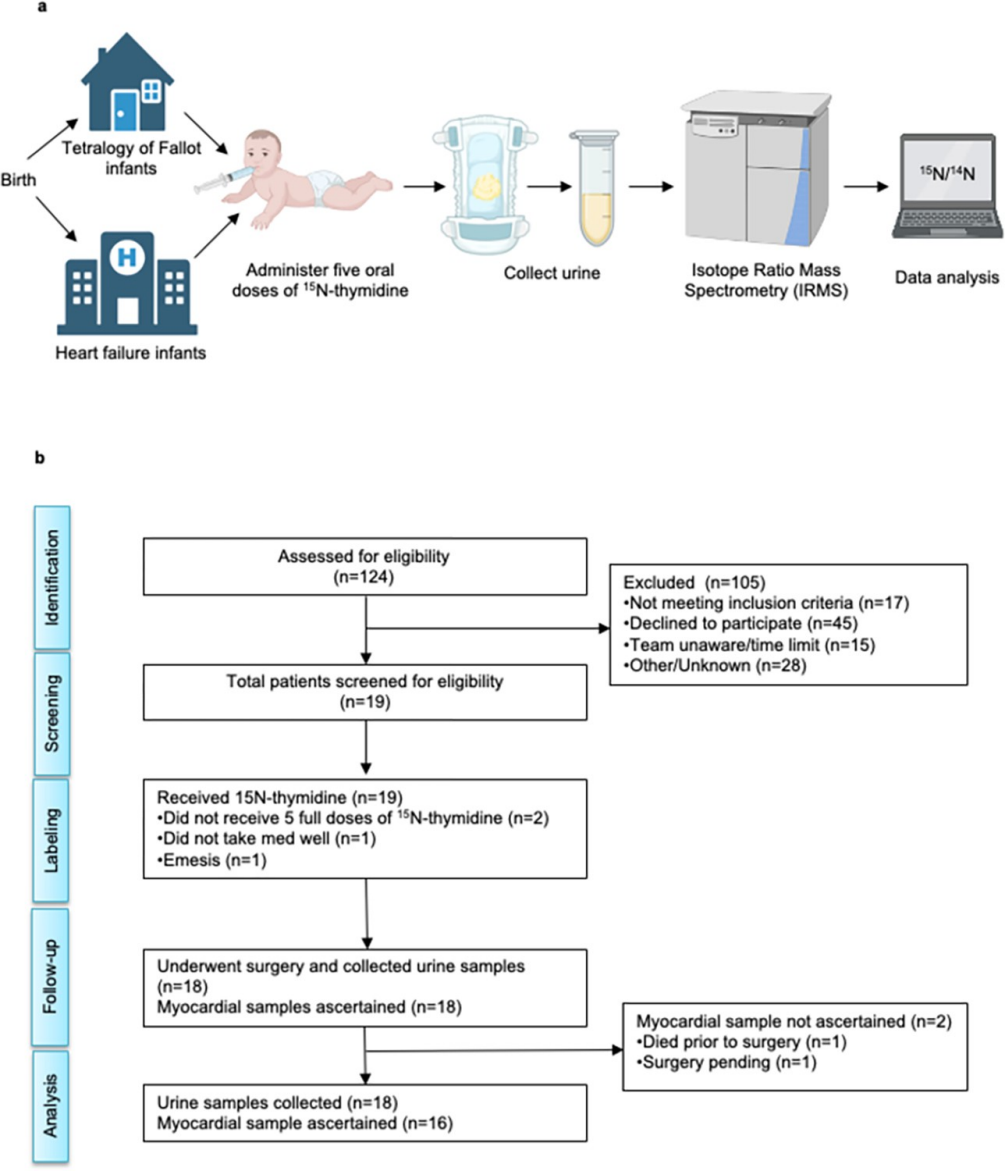
^15^N-thymdine administration and quantification in urine in infants. (a) Tetralogy of Fallot (ToF) infants were given ^15^N-thymidine at home with the exception of one patient and heart failure (HF) infants received ^15^N-thymidine at the hospital. Each infant received five oral doses of 50 mg/kg ^15^N-thymidine in individual single-dose syringes over the course of five consecutive days. Families filled out daily logs to record timing of doses, urine output, and any issues with dose administration. Cotton balls were placed in diapers and collected in specimen bags after infants voided. Cotton balls were collected up to 24 hours following the last dose. Cotton balls that dried during transportation and handling processes were discarded. Viable cotton balls were squeezed to express urine samples into separate 1.5 mL Eppendorf tubes and urine volumes were recorded. Samples were dried, crushed using forceps and weighed. Dried samples were analyzed by elemental analyzer isotope ratio mass spectrometry (IRMS). Atomic ratios of ^15^N/^14^N in each urine sample were expressed. (b) Enrollment and follow up of all study participants.

Because infants void frequently (up to once per hour) [17], we could utilize the recorded ^15^N-thymidine administration and urine collection times to examine temporal relationships between time of ^15^N-thymidine administration and excreted ^15^N/^14^N (%) concentration. **Fig 1A** shows the workflow of ^15^N-thymidine administration, urine collection, and analysis to quantify ^15^N-thymidine metabolism. A consort flowchart shows the enrollment and follow up of all study participants (**Fig 1B**).

**Fig 2:**
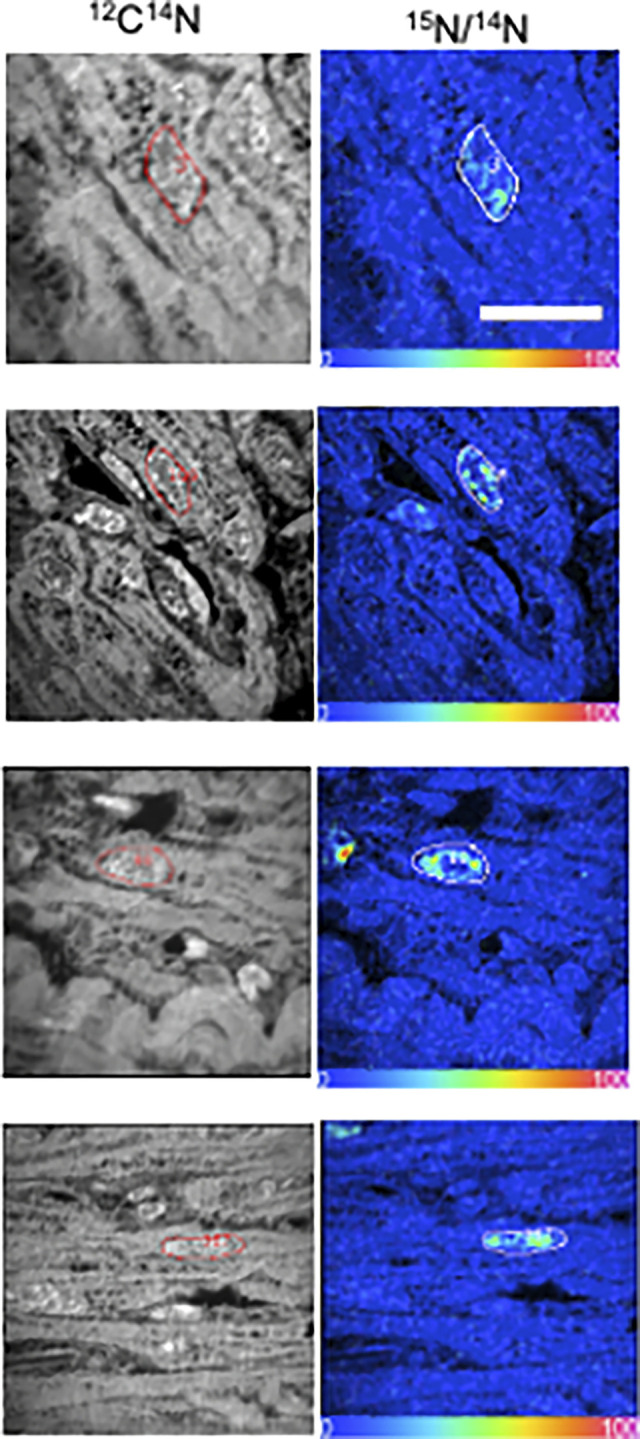
MIMS analysis of myocardial sample after oral ^15^N-thymidine labeling in an infant with ToF/PS. A 4-week-old human infant with ToF/PS received ^15^N-thymidine (given orally). Myocardium was resected at the time of ToF/PS repair at 7 months and analyzed by multiple-isotope imaging mass spectrometry (MIMS). The ^12^C^14^N secondary ion image is shown on the left. The quantitative isotope ratio, derived from the ^12^C^15^N and ^12^C^14^N measurements, is visually represented by the hue saturation image (HSI), whose scale is set so that 0 is the natural abundance and 100 is double of the natural abundance. Regions of interest (ROI), defined by the boundaries of nuclei, are outlined. A ^15^N/^14^N ratio above natural background identifies nuclei that underwent DNA replication during ^15^N-thymidine administration. All four outlined ROI are cardiomyocyte nuclei. Scale bar 10 μm. This patient was analyzed and published in Liu, Zhang, and Ammanamanchi et al 2019 (Ref. 4).

A heart muscle sample and MIMS results of the first pilot patient were available [4]. Four examples of marked cardiomyocyte nuclei demonstrate visual proof-of-principle (**Fig 2**).

**Fig 3:**
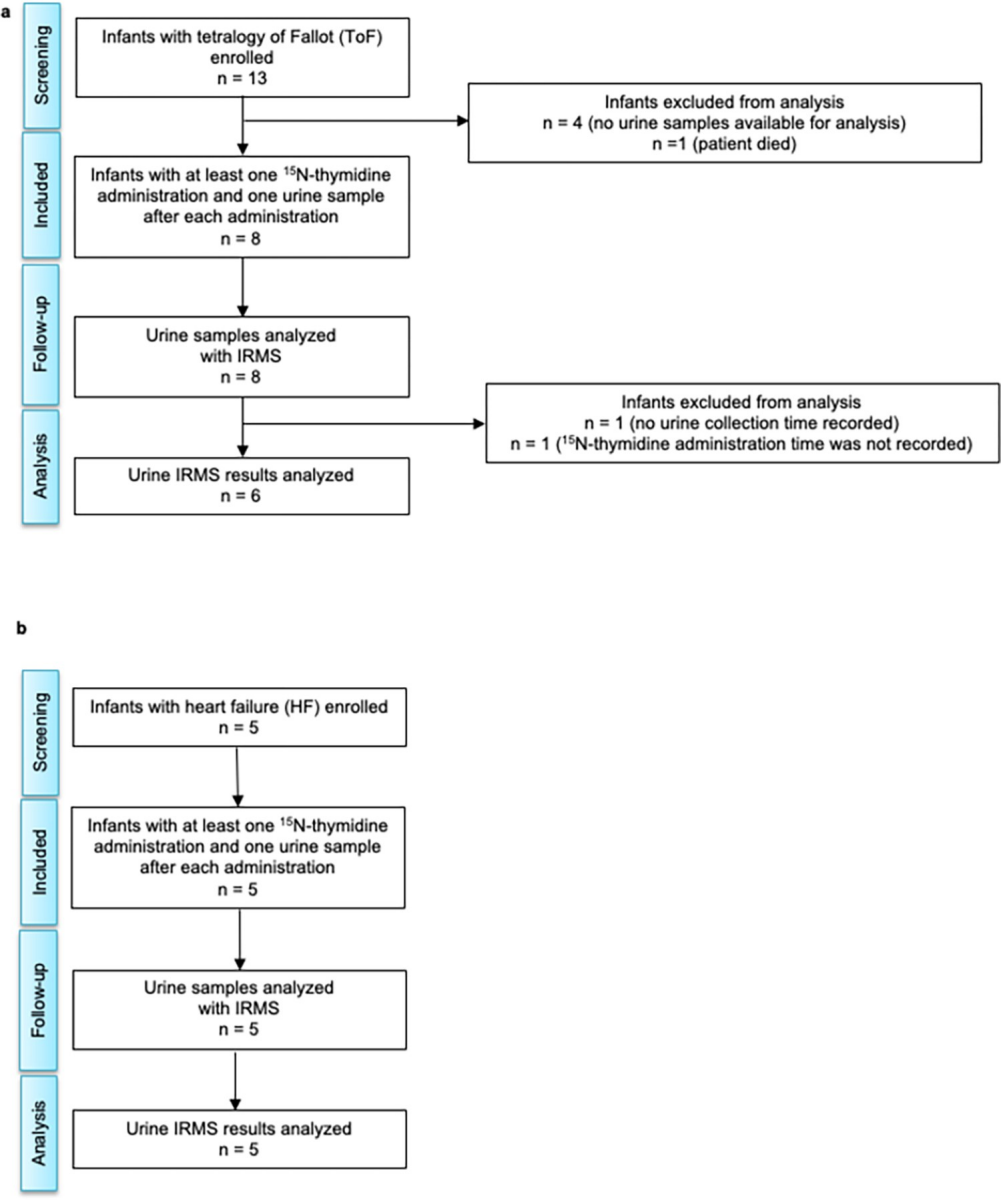
Recruitment and inclusion of results from 18 infants with heart disease. (a) Consort diagram shows that 13 infants with tetralogy of Fallot (ToF) were enrolled and received label; data from 6 infants was analyzed. (b) Consort diagram shows that 5 infants with heart failure (HF) were enrolled, received label, and urine was collected for analysis.

**Fig 4:**
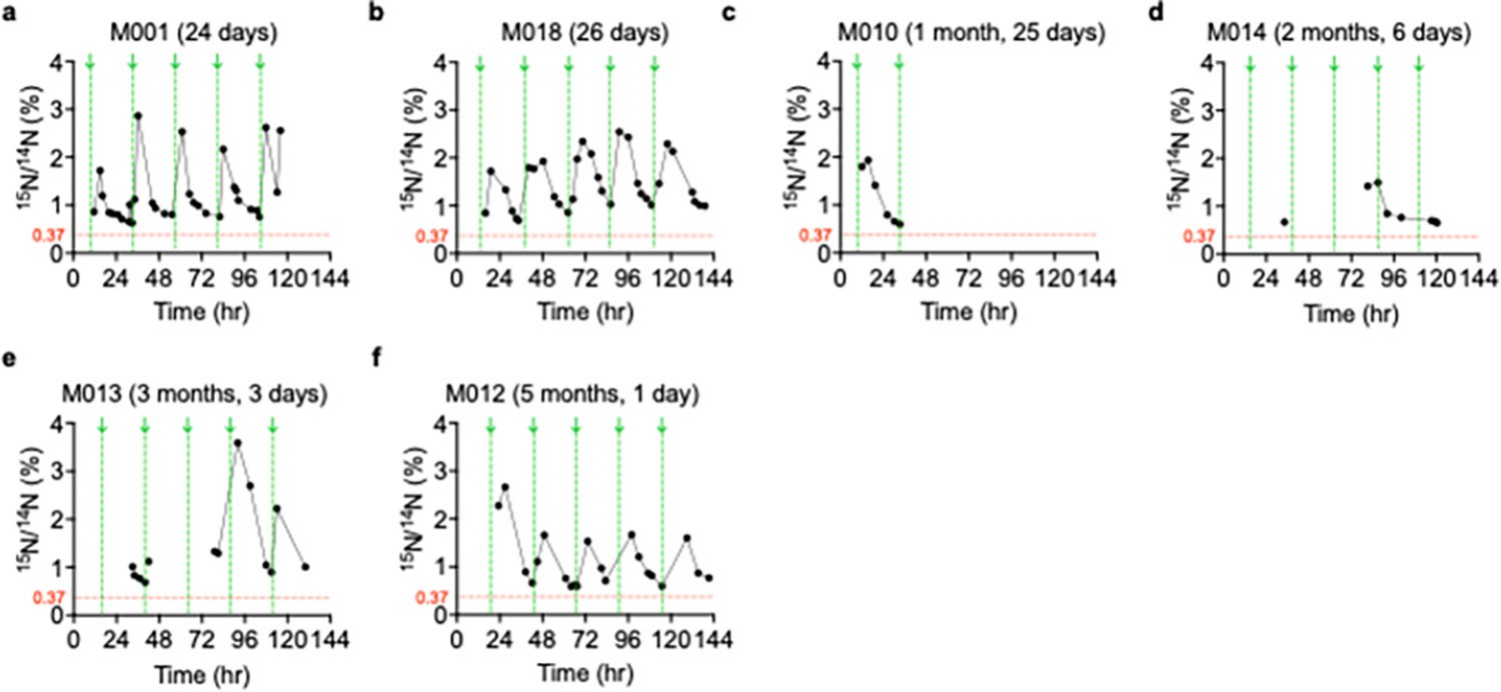
^15^N enrichment levels after ^15^N-thymidine administration show temporal patterns in infants with tetralogy of Fallot (ToF). Analysis of urine samples from six infants with ToF/PS who had received oral ^15^N-thymidine (50 mg/kg/day) was performed. Each graph has the patient study identifier and age at ^15^N-thymidine labeling indicated. Urine samples were extracted from diapers collected at home, dried, and subjected to Isotope Ratio Mass Spectrometry (IRMS) to quantify ^15^N/^14^N atomic ratios. The x-axes indicate timings of ^15^N-thymidine doses beginning with 00:00 hour of the day, and the Y-axes indicate the ^15^N/^14^N atomic ratios. Timing of ^15^N-thymidine administration is indicated with vertical green arrows (↓) and dotted lines. Each symbol (●) indicates one urine sample. Results of single samples are connected by solid black lines, unless > 24 hr apart. The threshold, *i*.*e*., the physiological ^15^N/^14^N atomic ratio, is indicated with a dotted red horizontal line.

**Fig 5:**
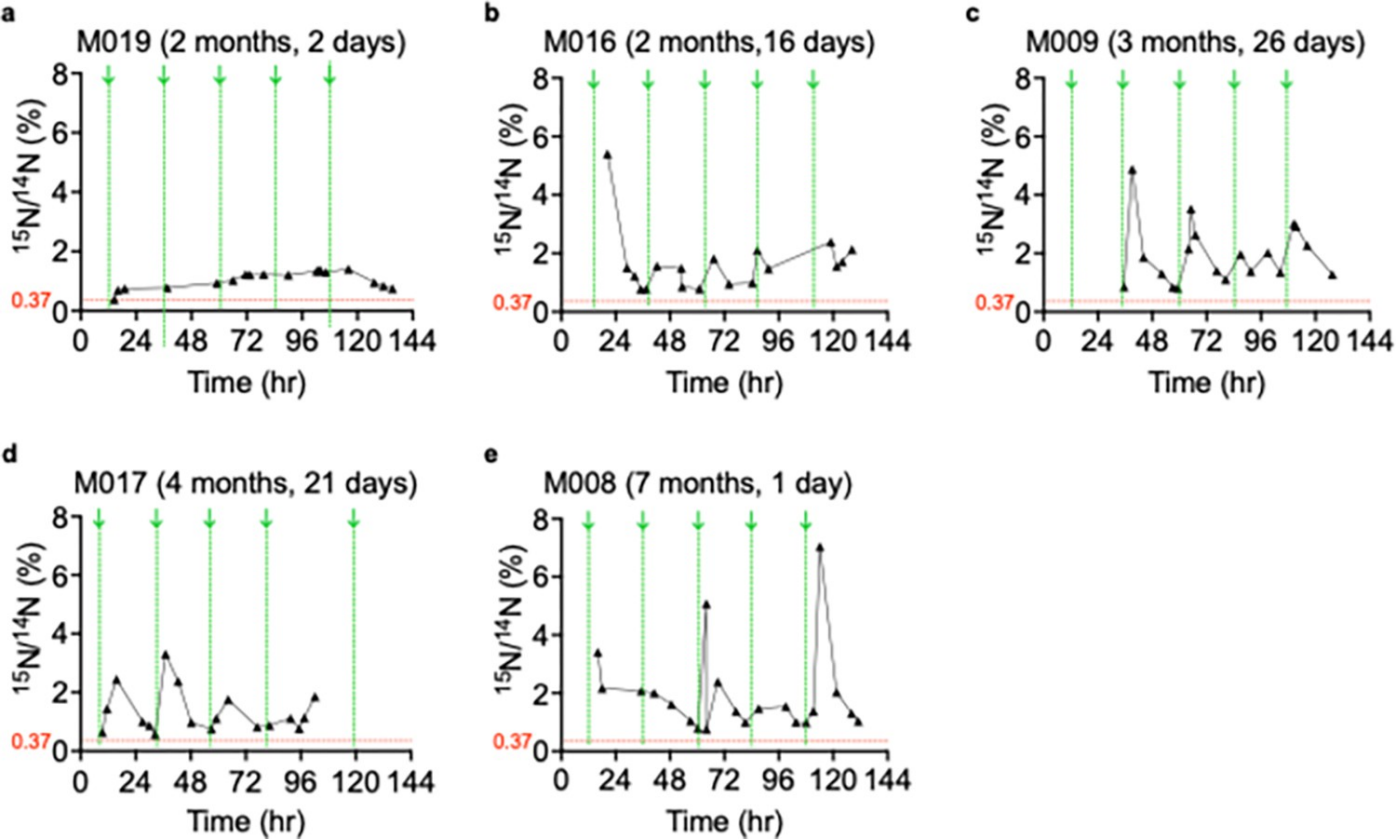
^15^N enrichment levels after ^15^N-thymidine administration show temporal patterns in infants with severe heart failure (HF). Analysis of urine samples from five infants on the wait list for heart transplantation, who had received oral ^15^N-thymidine (50 mg/kg/day) in the hospital was performed. Each graph has the patient code and age at ^15^N-thymidine labeling indicated. Urine samples were extracted from diapers collected in the hospital, dried, and subjected to Isotope Ratio Mass Spectrometry (IRMS) to quantify ^15^N/^14^N atomic ratios. The x-axes indicate timings of ^15^N-thymidine doses beginning with 00:00 hour of the day, and the Y-axes indicate the ^15^N/^14^N atomic ratios. Timing of ^15^N-thymidine administration is indicated with vertical green arrows (↓) and dotted lines. Each symbol (▲) indicates one urine sample. Results of single samples are connected by solid black lines, unless > 24 hr apart. The threshold, *i*.*e*., the physiological ^15^N/^14^N atomic ratio is indicated with a dotted red horizontal line.

**Fig 6:**
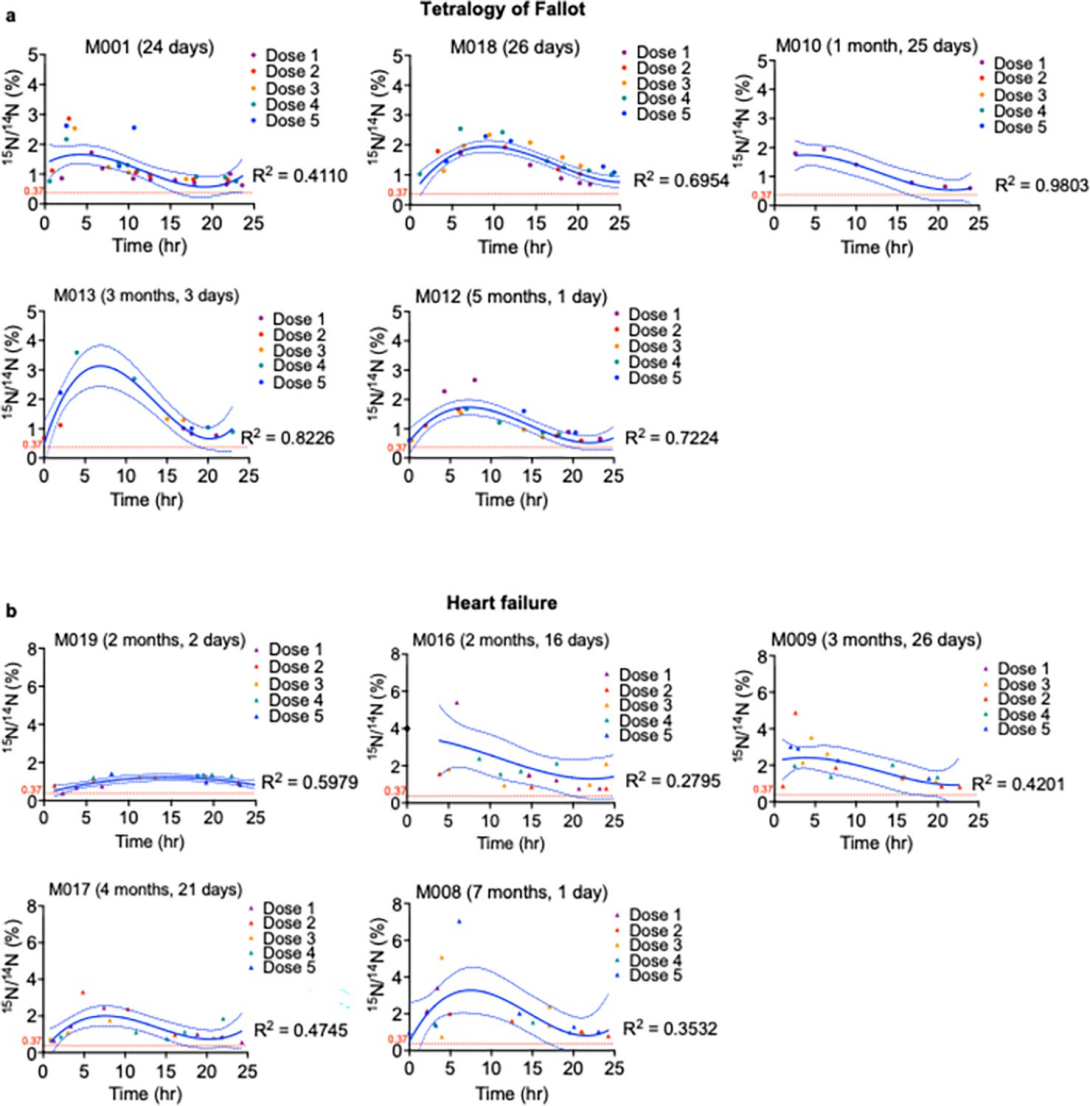
Aggregate data and curve fits of ^15^N urine enrichment from infants with tetralogy of Fallot (ToF) or heart failure (HF). Urine sample collection times shown in Figs 4 and 5 were normalized to time elapsed since the most recent ^15^N-thymidine administration, which was set to 0 hr. X-axes indicate time from ^15^N-thymidine administration to urine sampling. One patient (M014) was excluded from polynomial fitting due to insufficient urine data. Y-axes indicate ^15^N/^14^N (%) in urine. Each symbol represents one urine sample. The ^15^N/^14^N isotope ratio in percent was graphed over time elapsed since preceding ^15^N-thymidine dose administration. The normalization of urine collection times to each corresponding dose administration time allowed for overlapping the ^15^N/^14^N atomic ratios to achieve a higher data density for curve fitting. Urine samples corresponding to the same ^15^N-thymidine administration have the same color according to the legend. Centered third-degree polynomial functions, indicated in solid blue lines, were used as the best nonlinear regression model to fit the data. Dashed blue lines indicate 95% confidence intervals of the fits. The R^2^ values indicate the goodness of fit. The fits show that administered doses were sufficient to maintain the ^15^N/^14^N isotope ratios above baseline during the 5-day labeling period (a) Results from infants with ToF. (b) Results from infants with HF. The threshold, *i*.*e*., the physiological ^15^N/^14^N atomic ratio is indicated with a dotted red horizontal line.

**Fig 7:**
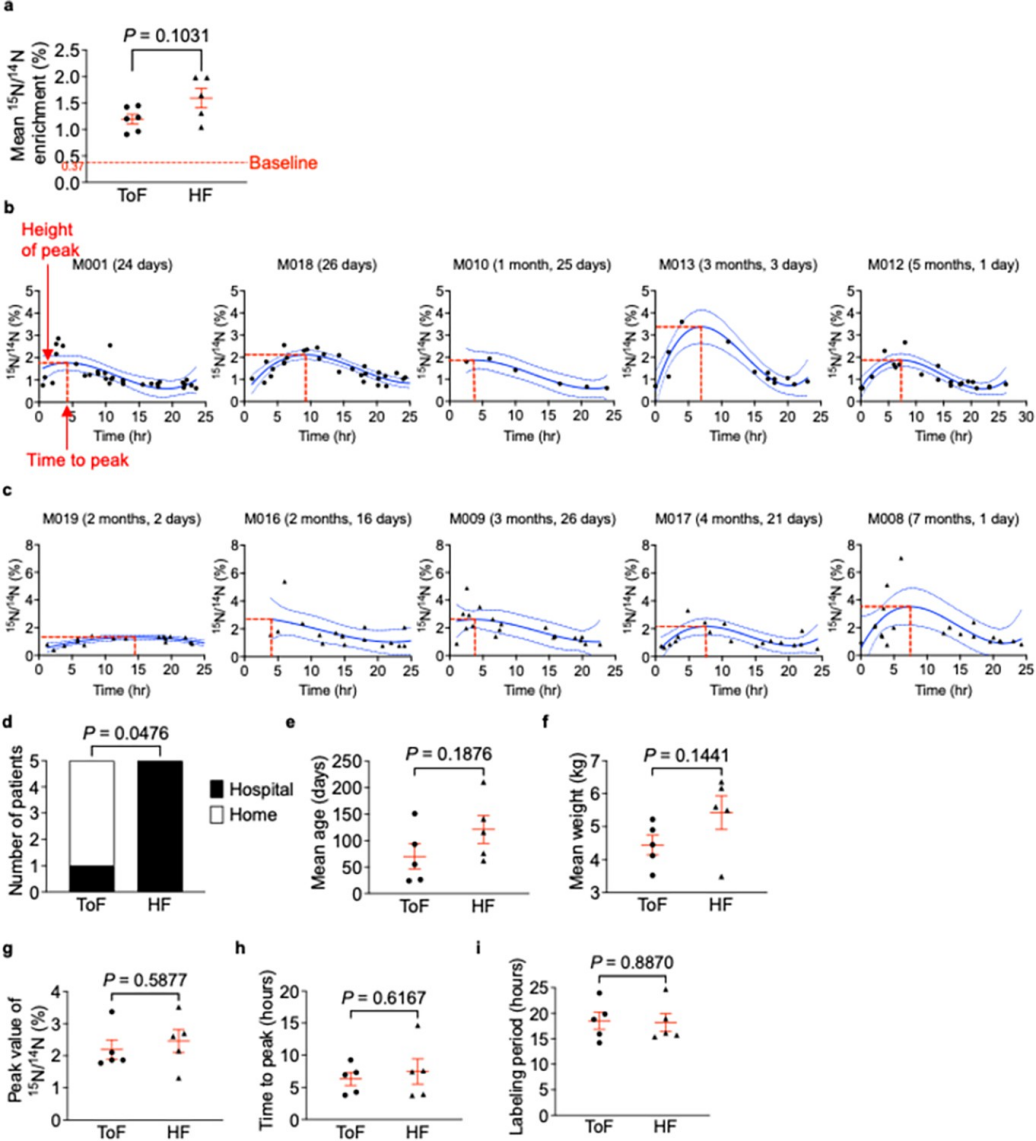
^15^N enrichment levels and temporal patterns in tetralogy of Fallot (ToF) and heart failure (HF) show similar absorption and elimination patterns. (a) Mean enrichment in ToF (n = 6) and HF (n = 5) patients is similar. Each symbol represents the mean of all urine values from one patient. (b, c) Data points were fit using a centered third-degree polynomial function (solid blue lines). Dashed blue lines indicate 95% confidence intervals. One patient (M014) was excluded from polynomial fitting due to insufficient urine data. X-axes indicate time from ^15^N-thymidine administration to urine sampling. Y-axes indicate ^15^N/^14^N (%) in urine. Comparisons were made between ToF and HF infants. (d) A statistically significant relationship exists between disease type (ToF / HF) and ^15^N-thymidine administration setting (home / hospital). Mean patient age (e) and weight (f) at label administration is similar in ToF and HF patients. (g) Peak ^15^N/^14^N atomic ratios are similar in patients with ToF and HF. (h) Time elapsed until peak enrichment is reached is similar in ToF and HF patients. (i) Labeling duration (time elapsed during which ^15^N/^14^N% > 0.37%) is similar in ToF and HF patients. (a, e-i) Statistical analysis: unpaired t-test with Welch’s correction. (d) Statistical analysis: Fisher’s exact test (b-i) ToF (n = 5); HF (n = 5).

A consort diagram shows the enrollment of patients with ToF and HF (**Fig 3**). Seven patients were excluded from analysis based on no or insufficient urine and/or ^15^N-thymidine records. Six ToF patients and five HF patients met the criteria necessary to be included in our subsequent analyses (**Figs 4–7**, **Tables 1 and 2**).

**Table 1:** Recruited patients with ToF.

Pt. identifier	Gender	Diagnosis	Age at label administration	Body weight at label administration (kg)	Number of urine samples collected	Location of labeling
M002	Female	ToF	23 DAYS	3.030	22	Home
M001*	Male	ToF	24 DAYS	4.120	32	Home
M018*	Male	ToF	26 DAYS	3.520	32	Home
M003	Male	ToF	1 MONTH, 1 DAY	3.800	0	Home
M006	Male	ToF/DORV	1 MONTH, 14 DAYS	2.580	0	Hospital
M010*	Male	ToF	1 MONTH, 25 DAYS	5.220	6	Home
M014*	Male	ToF	2 MONTHS, 6 DAYS	2.200	8	Hospital
M004	Female	ToF	2 MONTHS, 13 DAYS	3.090	0	Hospital
M007	Male	ToF	2 MONTHS, 27 DAYS	3.350	9	Home
M011	Male	ToF	3 MONTHS, 2 DAYS	5.050	25	Unknown
M013*	Male	ToF	3 MONTHS, 3 DAYS	4.450	13	Home
M005	Female	ToF	4 MONTHS, 6 DAYS	5.200	0	Hospital
M012*	Female	ToF	5 MONTHS, 1 DAY	4.900	21	Home

ToF, tetralogy of Fallot with pulmonary stenosis; ToF/DORV, double-outlet right ventricle with subpulmonary conus.

*Indicates patients that met criteria necessary to be included in subsequent analyses.

**Table 2:** Recruited patients with severe HF on the wait list for heart transplantation.

Pt. identifier	Gender	Diagnosis	Age at label administration	Body weight at label administration (kg)	Number of urine samples	Location of labeling
M019*	Male	HLHS	2 MONTH, 2 DAYS	3.490	18	Hospital
M016*	Female	DCM	2 MONTHS, 16 DAYS	5.500	18	Hospital
M009*	Male	DCM	3 MONTHS, 26 DAYS	6.170	19	Hospital
M017*	Male	DCM	4 MONTHS, 21 DAYS	5.600	18	Hospital
M008*	Female	DCM	7 MONTHS, 1 DAY	6.360	21	Hospital

DCM, dilated cardiomyopathy; HLHS, hypoplastic left heart syndrome.

*Indicates patients that met criteria necessary to be included in subsequent analyses

In general, the urine samples collected immediately following ^15^N-thymidine administrations showed increases in ^15^N enrichment followed by gradual decline until subsequent doses were administered. This sawtooth pattern is seen throughout the 5-day labeling period in both ToF infants (**Fig 4**) and HF infants (**Fig 5**). The temporal sequence of ^15^N-thymidine administration followed by increases and decreases of ^15^N enrichment in urine (**Fig 6**) suggested the hypothesis that there is a reproducible temporal relationship between ^15^N-thymidine administration and ^15^N enrichment. We considered a ^15^N/^14^N atomic ratio of 0.37% as an appropriate baseline in our infants because of previous physiological measurements [8]. To confirm this hypothesis, we measured baseline ^15^N abundance in control urine samples from infants who did not receive ^15^N-thymidine at 0.369 ± 0.001 ≈ 0.37% (n = 5, **S3 Table**), which consistent with previously published results. Consequently, all ^15^N enrichment was calculated as fold changes relative to the natural abundance of 0.37%. The mean ± SEM (%) of the ^15^N/^14^N atomic ratios in urine samples from ToF infants after ^15^N-thymidine administrations was 1.193 ± 0.092 (n = 6 infants), a 3.2-fold increase over baseline (**Fig 7A**). The samples from HF infants showed a mean ± SEM (%) ^15^N/^14^N atomic ratio of 1.590 ± 0.185 (n = 5 infants), a 4.3-fold increase over baseline (**Fig 7A**). The mean ^15^N enrichment between the two patient populations, ToF and HF, is not statistically different (P = 0.103, unpaired t-test with Welch’s correction; **Fig 7A).** All patients maintained ^15^N/^14^N atomic ratios above baseline for the duration of their 5-day labeling periods. Many urine samples per patient were 3-fold above baseline during the labeling period.

We compared the temporal ^15^N enrichment patterns between ToF and HF infants. To analyze the temporal relationship between ^15^N-thymidine administration and enrichment in urine, we overlaid sets of ^15^N/^14^N (%) urine collections-each set pertaining to the preceding dose administration. To this end, we set each ^15^N-thymidine administration time as 00:00 hr and plotted urine collection ^15^N/^14^N (%) values according to the elapsed time since the previous ^15^N-thymidine administration time. Thus, each patient had up to 5 datasets which allowed for greater data density. We applied a centered third-order polynomial fit to these data (**Figs 6 and 7**). One patient with insufficient urine data was excluded from polynomial fitting and subsequent analyses. Average patient age and weight at label administration and location of administration were compared between ToF and HF patients prior to analyses of temporal ^15^N enrichment patterns. Unpaired t-tests each with Welch’s correction indicated no age-related (ToF = 70 ± 24 (days), HF = 121 ± 26 (days), P = 0.188; **Fig 7E**) or weight-related (ToF = 4.442 ± 0.297 (kg), HF = 5.424 ± 0.510 (kg), P = 0.144; **Fig 7F**) differences between our patient populations. Fisher’s exact test was used to determine a statistically significant relationship between disease type (ToF, HF) and ^15^N-thymidine administration setting (home, hospital) (P = 0.048; **Fig 7D**).

We determined peak ^15^N enrichment values in ToF and HF infants, *i*.*e*., the fitted highest ^15^N/^14^N (%) value (**Fig 7B and 7C**). Infants with ToF showed a peak value of approximately 2.194 ± 0.299% (n = 5 patients, **Fig 7G**). Infants with HF showed peak value of approximately 2.459 ± 0.361% (n = 5 patients, **Fig 7G**). Peak enrichment levels were not significantly different between ToF and HF infants (P = 0.588, unpaired t-test with Welch’s correction; **Fig 7G**).

From the fitted curve, we also determined the time-to-peak, *i*.*e*., the duration from ^15^N-thymidine administration to the fitted highest ^15^N/^14^N (%) value (**Fig 7B and 7C**). The time to peak includes time for absorption into the blood stream, filtering in the kidney, and voiding, as well as lag time for ascertainment of urine samples from diapers by the caregivers or hospital staff. Because time to peak may be influenced by potentially differing metabolic rates, we compared ToF and HF. Infants with ToF showed a time to peak of approximately 6.302 ± 1.017 hours (n = 5 patients, **Fig 7H**). Infants with HF showed a similar time to peak of approximately 7.475 ± 1.976 hours (n = 5 patients, **Fig 7H**). The time to peak between ToF and HF infants was not significantly different (P = 0.617, unpaired t-test with Welch’s correction; **Fig 7H**). Taken together, the urine samples collected within the first 6 hours following label administration showed the greatest fold-increases in ^15^N enrichment. It took relatively the same amount of time post administration to reach peak ^15^N enrichment in both ToF and HF infants.

We then examined the labeling period from the fitted results. The labeling period was defined as the period of time during which the lower 95% CI of the third-degree polynomial fit curve > physiological baseline (0.37%). The mean labeling period and range of variability was 18.540 ± 1.686 for ToF patients and 18.190 ± 1.751 for HF patients (**Fig 7I**). Infants with ToF and HF did not differ significantly in their labeling period lengths (P = 0.887, unpaired t-test with Welch’s correction; **Fig 7I**). This analysis showed that the administered doses were sufficient to raise the ^15^N/^14^N atomic values above baseline for the majority of the 5-day labeling period in ToF and HF infants. **Table 3** summarizes the demographic and temporal characteristics of the two groups of patients who received the ^15^N-thymidine label. We concluded that once-daily oral administration of 50 mg/kg provides sufficient ^15^N enrichment in both ToF and HF infants to label cardiomyocyte nuclei in S-phase, which has a reported duration of approximately 10 hr [20].

**Table 3:** Summary of comparison between ToF and HF infants.

	ToF	HF (DCM, HLHS)	Statistical significance (*P*)
Number of patients analyzed	5	5	NA
Administration in hospital	1 (20%)	5 (100%)	0.048
Mean age at administration (days)	70 ± 24	121 ± 26	0.188
Mean weight at administration (kg)	4.442 ± 0.297	5.424 ± 0.510	0.144
Peak ^15^N/^14^N atomic ratio (%)	2.194 ± 0.299	2.459 ± 0.361	0.588
Time to peak (hours)	6.302 ± 1.017	7.475 ± 1.976	0.617
Labeling period (hours)	18.540 ± 1.686	18.190 ± 1.751	0.887

This table corresponds to Fig 7(B)–7(I). Fisher’s exact test was used to determine administration in hospital p-value. Subsequent quantitative results are given as mean ± SEM. Statistical significances of mean differences were tested with unpaired t-test with Welch’s correction. n = 5 ToF infants; n = 5 HF infants. ToF, tetralogy of Fallot; DCM, dilated cardiomyopathy; HLHS, Hypoplastic left heart syndrome.

## Discussion

The oral administration of ^15^N-thymidine is convenient and sufficient to detect cardiomyocyte generation in infants (**Fig 2**) [4], however, the temporal patterns of ^15^N enrichment in the body remained unknown. Here, we present a systematic study of ^15^N-elimination in urine as a proxy for circulating, *in vivo* ^15^N enrichment. Peak ^15^N-elimination occurs within a short interval after administration, suggesting rapid bioavailability after oral dosing. This is followed by a period of sustained enrichment for approximately 18 hr. This bioavailability pattern after oral administration provides advantages as compared to traditional intravenous bolus administration of labels, which produces a rapid rise and fall of blood concentrations [21]. S-phase in eukaryotic cell lines has a duration of > 8 hr and in primary peripheral blood lymphocytes ~ 12 hr [20], therefore most cells that are in S-phase during the 5 day-block of label administration are likely to incorporate ^15^N-thymidine at sufficient levels for detection by MIMS. Collectively, the presented results demonstrated that 50 mg/kg daily of ^15^N-thymidine administered orally to infants was able to effectively increase the bioavailability of ^15^N-thymidine, which can be used to detect and quantify proliferating cells.

Although the presented results validate the oral administration of ^15^N-thymidine for read-out with secondary ion mass spectrometry (SIMS, **Fig 2**), the findings are relevant for other mass spectrometry techniques, whether for imaging or not. As such, the presented findings could be useful for Matrix-Assisted Laser Desorption/Ionization (MALDI) and time-of-flight secondary ion mass spectrometry (TOF-SIMS) imaging [22, 23].

This study also addresses theoretical differences in administration between caregivers and hospital staff. For patients who received administration at home, caregivers were responsible for all research related tasks, i.e., they administered the ^15^N-thymidine label doses, collected urine samples, and recorded administration and sampling times. As such, the design and conduct of this study had to account for the limitations of what caregivers are willing to do and can do. Although we prepared caregivers for these research tasks with instructions and provided formatted paper data entry logs, and performed check-ins by telephone, it must be clearly stated that this part of the data was not obtained by trained clinical research coordinators. In contrast to most patients with ToF, patients with HF were admitted to the hospital and so the data was recorded by medical professionals. This offered the opportunity to compare and cross-validate, which showed similar results with at-home and in-hospital administration. As such, our study validates that at-home administration of ^15^N-thymidine is a useful clinical research approach for marking cellular renewal and regeneration.

In conclusion, our results define the bioavailability of oral ^15^N-thymidine administration using urinary elimination as a proxy. Urinary elimination is useful to confirm compliance with administration and adequate absorption for infants labeled at home and can be used to define a labeling period that can facilitate the calculation of proliferation rates.

## Supporting information

S1 Fig Comparing different fitting functions for aggregate data of ^15^N urine enrichment from infants with ToF or HF. (TIFF)

S1 Table IRB approval summary for observational study. (PDF)

S2 Table Primary data for examining appropriateness of third-degree polynomial fitting. (PDF)

S3 Table Primary data for negative control urine measurements. (PDF)
