# Supplementary figures and images for: Elimination of 15N-thymidine after oral administration in human infants
Source: PLoS One. 2024 Jan 25;19(1):e0295651. doi: 10.1371/journal.pone.0295651 (PMC10810423; doi:10.1371/journal.pone.0295651)

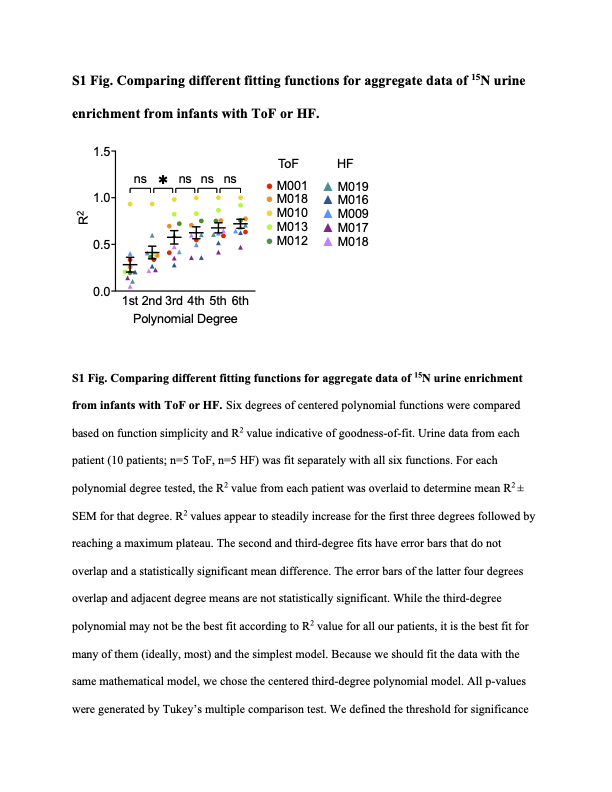

Supplement: S1 Fig — (TIFF) [file pone.0295651.s001.tiff]
