# Supplementary material for: Elimination of 15N-thymidine after oral administration in human infants
Source: PLoS One. 2024 Jan 25;19(1):e0295651. doi: 10.1371/journal.pone.0295651 (PMC10810423; doi:10.1371/journal.pone.0295651)
Supplement: S1 Table — (PDF) [file pone.0295651.s002.pdf]

S1 Table. IRB Approval Summary for observational study

**OSIRIS IRB Approval Summary – PRO14100518 – A Pilot Study Quantifying New Heart Muscle Cells in Pediatric Patients**			
IRB #	*Approval Date*	*Expiration*	*Document type/comments*
PRO14100518	7/23/2015	7/8/2016	Initial approval
REN16040245/PRO14100518	5/12/2016	5/11/2017	Renewal
REN17030233/PRO14100518	4/4/2017	4/3/2018	Renewal
REN18030120/PRO14100518	3/19/2018	4/3/2019	Renewal
PRO14100518		4/4/2019	OSIRIS Close-out/transition to PittPro

**PittPro IRB Approval Summary – STUDY19030250 (formerly PRO14100518) – A Pilot Study for Quantifying New Heart Muscle Cells**			
IRB #	*Approval Date*	*Expiration*	*Document type/comments*
STUDY19030250	4/2/2019	None	Initial Study – Conversion to Pitt Pro. Released from continuing review

Legend: Summary of approval and renewal history for protocol entitled "A Pilot Study Quantifying New Heart Muscle Cells in Pediatric Patients" (OSIRIS and PittPro PRO14100518).
