# Supplementary material for: Elimination of 15N-thymidine after oral administration in human infants
Source: PLoS One. 2024 Jan 25;19(1):e0295651. doi: 10.1371/journal.pone.0295651 (PMC10810423; doi:10.1371/journal.pone.0295651)
Supplement: S2 Table — (PDF) [file pone.0295651.s003.pdf]

S2 Table. Primary data for examining appropriateness of third-degree polynomial fitting

Degree of Polynomial Function	Mean R^2 ± SEM
1st	0.283 ± 0.079
2nd	0.413 ± 0.067
3rd	0.576 ± 0.071
4th	0.624 ± 0.064
5th	0.677 ± 0.054
6th	0.721 ± 0.048

Legend: Comparing different degrees of polynomial fitting functions for aggregate data of ^{15}N enrichment in urine from infants with ToF/PS or HF. This table corresponds to Fig S1.
