# Supplementary material for: Elimination of 15N-thymidine after oral administration in human infants
Source: PLoS One. 2024 Jan 25;19(1):e0295651. doi: 10.1371/journal.pone.0295651 (PMC10810423; doi:10.1371/journal.pone.0295651)
Supplement: S3 Table — (PDF) [file pone.0295651.s004.pdf]

S3 Table. Primary data for negative control urine measurements.

**Sample ID**	**Well#**	**Amount**	**Ampl 28**	**%N**	**%C**	**C:N**		d15N	**d13C**		15N/14N (%)	**13C/12C**
NTCl 1	A2	2.6	7372	11.2	16.2	1.4		1.24	-19.11		0.368055824	0.010966395
NTCl 2	A3	2.2	6692	12.2	23.4	1.9		2.811	-20.66		0.368633324	0.010949077
NTCl 5	A4	4.5	16177	13.7	28.2	2.1		5.103	-19.36		0.369475863	0.01096351
NTCl 6	A5	2	2134	4.3	6.7	1.6		4.982	-19.60		0.369431383	0.010960928
NTCl 7	A6	1.6	5047	12.4	21.2	1.7		2.722	-20.67		0.368600607	0.010948909
										AVG	0.3688394	
AIR	0.003676						d15N Error on standards for this run	0.15		STD	0.000606014	
PDB	0.01118							(1 sigma)				
											0.369 +/- 0.001	

Legend: IRMS measurements were performed on urine samples from five infants.
